# Adding abiraterone to androgen deprivation therapy in men with metastatic hormone-sensitive prostate cancer: A systematic review and meta-analysis

**DOI:** 10.1016/j.ejca.2017.07.003

**Published:** 2017-10

**Authors:** Larysa H.M. Rydzewska, Sarah Burdett, Claire L. Vale, Noel W. Clarke, Karim Fizazi, Thian Kheoh, Malcolm D. Mason, Branko Miladinovic, Nicholas D. James, Mahesh K.B. Parmar, Melissa R. Spears, Christopher J. Sweeney, Matthew R. Sydes, NamPhuong Tran, Jayne F. Tierney, the STOPCaP Abiraterone Collaborators

**Affiliations:** a MRC Clinical Trials Unit at UCL, Aviation House, 125 Kingsway, London WC2B 6NH, UK; b Salford Royal NHS Foundation Trust, Salford, UK; c Gustave-Roussy, University of Paris Sud, 114 Rue Edouard Vaillant, Villejuif 94800, France; d Janssen Research & Development, San Diego, CA, USA; e Cardiff University, School of Medicine, Cardiff, UK; f Institute of Cancer and Genomic Sciences, University of Birmingham, Birmingham, UK; g Queen Elizabeth Hospital, Birmingham, UK; h Dana-Farber Cancer Institute, Harvard Medical School, Boston, MA 02215, USA; i Janssen Research & Development, Los Angeles, CA, USA

**Keywords:** Prostate cancer, Metastases, Abiraterone, Systematic review, Meta-analysis, Androgen deprivation therapy

## Abstract

**Background:**

There is a need to synthesise the results of numerous randomised controlled trials evaluating the addition of therapies to androgen deprivation therapy (ADT) for men with metastatic hormone-sensitive prostate cancer (mHSPC). This systematic review aims to assess the effects of adding abiraterone acetate plus prednisone/prednisolone (AAP) to ADT.

**Methods:**

Using our framework for adaptive meta-analysis (FAME), we started the review process before trials had been reported and worked collaboratively with trial investigators to anticipate when eligible trial results would emerge. Thus, we could determine the earliest opportunity for reliable meta-analysis and take account of unavailable trials in interpreting results. We searched multiple sources for trials comparing AAP plus ADT versus ADT in men with mHSPC. We obtained results for the primary outcome of overall survival (OS), secondary outcomes of clinical/radiological progression-free survival (PFS) and grade III–IV and grade V toxicity direct from trial teams. Hazard ratios (HRs) for the effects of AAP plus ADT on OS and PFS, Peto Odds Ratios (Peto ORs) for the effects on acute toxicity and interaction HRs for the effects on OS by patient subgroups were combined across trials using fixed-effect meta-analysis.

**Findings:**

We identified three eligible trials, one of which was still recruiting (PEACE-1 (NCT01957436)). Results from the two remaining trials (LATITUDE (NCT01715285) and STAMPEDE (NCT00268476)), representing 82% of all men randomised to AAP plus ADT versus ADT (without docetaxel in either arm), showed a highly significant 38% reduction in the risk of death with AAP plus ADT (HR = 0.62, 95% confidence interval [CI] = 0.53–0.71, p = 0.55 × 10^−10^), that translates into a 14% absolute improvement in 3-year OS. Despite differences in PFS definitions across trials, we also observed a consistent and highly significant 55% reduction in the risk of clinical/radiological PFS (HR = 0.45, 95% CI = 0.40–0.51, p = 0.66 × 10^−36^) with the addition of AAP, that translates to a 28% absolute improvement at 3 years. There was no evidence of a difference in the OS benefit by Gleason sum score, performance status or nodal status, but the size of the benefit may vary by age. There were more grade III–IV acute cardiac, vascular and hepatic toxicities with AAP plus ADT but no excess of other toxicities or death.

**Interpretation:**

Adding AAP to ADT is a clinically effective treatment option for men with mHSPC, offering an alternative to docetaxel for men who are starting treatment for the first time. Future research will need to address which of these two agents or whether their combination is most effective, and for whom.

## Highlights

• First systematic review of abiraterone acetate plus prednisone/prednisolone (AAP) in hormone-sensitive prostate cancer.
• Results based on 2201 men (82% of all eligible), including unreported analyses.
• Adding AAP to hormones substantially improves survival and progression-free survival.
• Benefits may vary with age, but not nodal or performance status, or Gleason score.
• No increased mortality with AAP, but more cardiac, vascular and hepatic toxicity.

## 1 Introduction

For decades, the standard of care for men with metastatic, hormone-sensitive prostate cancer (mHSPC) has been castration, also called androgen deprivation therapy (ADT). This is achieved either surgically with bilateral orchiectomy or medically with luteinising hormone–releasing hormone (LHRH) agonists/antagonists [1], [2]. ADT produces responses in up to 95% of men, but it is not curative and disease progresses in virtually all patients [1]. Numerous randomised controlled trials (RCTs) have evaluated, or are currently evaluating, the addition of other therapies to ADT. These include, cytotoxic chemotherapy, radium-223 and next generation androgen receptor axis inhibitors, including abiraterone acetate and enzalutamide [3], [4], [5], [6]. There will be a need to synthesise the results of these trials to determine reliably which treatments are most effective. Thus, we are conducting a series of systematic reviews under the auspices of the Systemic Treatment Options for Prostate Cancer (STOPCaP) collaboration.

Most systematic reviews use aggregate data (AD) from publications and are retrospectively planned. Consequently, they can suffer from reporting biases, be unreliable and lag behind therapeutic developments, thus failing to influence ongoing or new trials. Therefore, we have developed a novel framework for adaptive meta-analysis (FAME) [7] to determine the earliest opportunity for reliable AD meta-analysis. FAME is a prospective and collaborative approach that takes all relevant trials into account, whether published, unpublished or ongoing, and is therefore more responsive to emerging trial results. FAME highlighted that key trials investigating the addition of abiraterone acetate plus prednisone/prednisolone (AAP) to ADT in mHSPC were due to report results, triggering the current systematic review and meta-analysis. The primary aim was to assess the effects of AAP in combination with ADT on overall survival (OS), progression and acute treatment-related toxicity on men with mHSPC. Our secondary aim was to investigate whether any effect of AAP varies across different subgroups of men.

## 2 Methods

Methods for this systematic review and meta-analysis were pre-specified in a protocol (PROSPERO registration: CRD42017058300) [8], and the review was conducted in accordance with the Preferred Reporting Items for Systematic Reviews and Meta-analyses (PRISMA) guidelines [9].

### 2.1 Framework for adaptive meta-analysis (FAME)

We have developed and successfully piloted FAME in systematic reviews of docetaxel and bisphosphonates in metastatic and non-metastatic prostate cancer [10]. In this review, we have adopted the key principles of FAME [7], which are to (1) start the review process before all, or indeed most, trials have completed; (2) identify all published, unpublished and ongoing eligible trials; (3) work collaboratively with trial teams to develop a detailed picture of how information and results are likely to accumulate from trials; (4) predict the feasibility and timing of a reliable meta-analysis (based on a large proportion of eligible patients being included, power to detect a clinically meaningful effect and reasonable follow-up); (5) take account of trials that have not yet completed/reported in interpreting results and (6) determine if an update is needed and whether it should be based on AD or individual participant data (IPD).

### 2.2 Trial eligibility

RCTs were eligible if they compared ADT plus AAP versus ADT in men with mHSPC. Trials including other additional agents (e.g. docetaxel or radiotherapy [RT]) were also eligible, provided the additional treatment was given in both treatment and control arms. Those that included additional treatments on the control arm only were ineligible. Trials that randomised men who had failed first-line hormone therapy for metastatic prostate cancer or men with castrate-refractory prostate cancer were also ineligible.

### 2.3 Trial identification

As part of the wider STOPCaP project, we regularly and systematically searched a number of trial sources to identify all published, unpublished and ongoing trials in men with mHSPC. This provides a comprehensive and up-to-date database of all RCTs eligible for all of our STOPCaP systematic reviews. We also requested regular updates from relevant trial teams on the status and reporting plans. Trials pertinent to this particular review of AAP were identified as part of this broader search.

With no restriction on language, LHMR, SB and CLV searched MEDLINE, Embase, clinicaltrials.gov and the Cochrane Central Register of Controlled Trials to May 2017, using database-specific search strategies [22], [23] (Web Appendix 1). We also searched proceedings from relevant conferences, such as the American Society of Clinical Oncology, the European Society for Medical Oncology, the European Cancer Organisation, the American Urological Association and the European Association of Urology to May 2017 (Web Table 1). In addition, reference lists of review articles and bibliographies of identified trial reports were screened for further eligible trials.

Once duplicates were removed, all relevant records were independently assessed for eligibility by three reviewers (LHMR, SB and CLV). Full articles or protocols, where available, were obtained for records deemed potentially eligible. All three reviewers agreed the final set of eligible RCTs and determined which of these were relevant specifically to this review of AAP. Collaborators, including representatives from the manufacturers of abiraterone acetate (Janssen), were also asked to review, and where possible, supplement our provisional list of eligible trials.

### 2.4 Outcomes

The primary outcome was OS, defined as the time from randomisation to death from any cause. The secondary outcomes were progression-free survival (PFS), defined as the time from randomisation to first evidence of symptomatic clinical progression or radiological progression or death (excluding biochemical (prostate-specific antigen [PSA]) progression) and failure-free survival (FFS), defined as time to first biochemical (PSA), clinical or radiological progression. Further secondary outcomes were grade III–IV and grade V toxicity (as defined in each trial). These outcomes were prospectively chosen because they were listed in the trial protocols, and their definitions are sufficiently similar to allow them to be combined across trials.

### 2.5 Data collection

For eligible trials, and for men with metastatic disease, we sought information on the following: trial accrual period, number of patients, patient age, PSA, performance status, T and N category, location of metastases, disease history, Gleason sum score and hormone therapy from publications, protocols and directly from investigators. We also sought results overall for OS, PFS and FFS as well as by patient subgroups, defined by age, Gleason sum score, nodal status, performance status, type of hormone therapy, location of metastases and disease history. We also requested results for all grades of acute treatment toxicity, as collected within trials, and for the main grade III–IV categories, which we tried to match across trials.

To assess the risk of bias of included trials, based on the outcome of OS, we also sought information on the method of randomisation sequence generation, allocation concealment, blinding of participants, personnel and outcome assessment, completeness of outcome data and whether all key outcomes were reported/available.

### 2.6 Planning the meta-analysis

Initial searches identified three eligible trials (Table 1). In 2016, through contact with investigators, we anticipated that by 2017, one trial, PEACE-1 (NCT01957436) would still be recruiting patients, but the two other trials (LATITUDE (NCT01715285) and STAMPEDE (NCT00268476)) would be reporting results [11], [12]. Based on recruitment information available at that time, we predicted that the latter two trials would represent over 70% of metastatic patients eligible for this comparison. Given that these trials were large and adequately powered and that PEACE-1 is unlikely to produce results before 2020, this provided the trigger to initiate an early systematic review and meta-analysis. However, we also planned to take into account the potential impact of the results of PEACE-1.

**Table 1:** Characteristics of studies eligible trials.

Trial	Accrual dates	Number of M1 patients	*De novo* or relapsed M1?	Control	Treatment	Median age (range)	Gleason score of 8–10 (%)	Performance status 0–1 (%)	Median follow-up (survival)
STAMPEDE [12] (Arm A versus arm G) M1 patients only	11/2011–01/2014	1002	*De novo* (95%) or relapsed after local therapy (5%)	ADT (LHRH agonist or antagonist or orchiectomy)	ADT + abiraterone (1000 mg/d) + prednisone (5 mg/d)	67 (62–72)	737 (74%)	988 (97%)	41 months

LATITUDE [11]	02/2013–12/2014	1199	*De novo*	ADT (LHRH agonists or orchiectomy)	ADT + abiraterone (1000 mg/d) + prednisone (5 mg/d)	67 (33–92)	1170 (98%)	1157 (96%)	30.4 months

PEACE-1^a^ (NCT01957436) (patients not receiving docetaxel in addition to ADT)	11/2013–to date	≈476 expected	*De novo*	ADT (LHRH agonist or antagonist or orchiectomy)	ADT + abiraterone (1000 mg/d) + prednisone (10 mg/d)	Not yet available	Not yet available	Not yet available	Not yet available
				ADT (LHRH agonist or antagonist or orchiectomy) + radiotherapy (74 Gy, 37 fractions)	ADT + abiraterone (1000 mg/d) + prednisone (10 mg/d) + radiotherapy (74 Gy, 37 fractions)				

PEACE-1^b^ (NCT01957436) (patients receiving docetaxel in addition to ADT)	11/2015–ongoing	Target ≈650 (≈300+ accrued to date)		ADT (LHRH agonist or antagonist or orchiectomy) + docetaxel^c^ (75 mg/m^2^ q 21 days; 6 cycles)	ADT + docetaxel^c^ + abiraterone (1000 mg/d) + prednisone (10 mg/d)	Not yet available	Not yet available	Not yet available	Not yet available
				ADT (LHRH agonist or antagonist or orchiectomy) + docetaxel^c^ (75 mg/m^2^ q 21 days; 6 cycles) + radiotherapy (74 Gy, 37 fractions)	ADT + docetaxel^c^ + abiraterone (1000 mg/d) + prednisone (10 mg/d) + radiotherapy (74 Gy, 37 fractions)				

ADT, androgen deprivation therapy; LHRH, luteinising hormone–releasing hormone.

a Patients randomised to PEACE-1, who have not received docetaxel in addition to ADT are eligible for this comparison.

b Patients randomised to PEACE-1, who have received docetaxel in addition to ADT will be eligible for a subsequent comparison of the systematic review (PROSPERO CRD42017058300).

c Docetaxel use is left to the investigator's discretion (stratification factor).

### 2.7 Measuring treatment effects and conducting the meta-analysis

For time-to-event outcomes (OS and PFS), the hazard ratios (HRs) and associated statistics from each trial were sought directly from investigators. These were combined using the fixed-effect model to give HRs representing the overall risk of an event on AAP compared with ADT [13]. For toxicity, the number of grade III–IV toxicities and the number of patients were sought directly from investigators. These were used to calculate Peto odds ratio (Peto OR) estimates of treatment effect [13] because this measure performs well when event rates are low [14]. Peto OR estimates for the individual trials were pooled across trials, using the fixed-effect model, to give ORs representing the risk of an event on AAP versus ADT. Chi-square tests and the I^2^ statistic were used to assess the statistical heterogeneity [15].

We also planned to investigate whether any effect of treatment on OS was consistent across patient subgroups including age (as defined in the trials), performance status (0, 1+), nodal status (N0, N+), Gleason sum score (<8, ≥8), type of hormone therapy (orchiectomy, LHRH-agonist or LHRH-antagonist), location of metastases (bone, bone and soft tissue, or soft tissue only) and disease history (*de novo* metastatic disease or relapsed after prior local therapy with curative intent). If there were insufficient numbers of men within any of these subgroups, we either combined them to achieve groups of a reasonable size or did not perform subgroup analyses. If categories were incompatible across trials, we worked to re-categorise the subgroups (e.g. performance status 0, 1+ instead of 0–1, 2) and requested trial subgroup analysis results based on these new categories. For subgroup variables with two categories, an interaction HR was calculated from the ratio of HRs derived from each trial's subgroup analyses (e.g. the HR for Gleason score <8 divided by the HR for Gleason score ≥8). For subgroup variables with three ordered categories, interaction HRs were estimated using a weighted linear regression of subgroup HRs, with the assumption that the error variances were known. These interaction HRs were then combined across trials using a fixed-effect meta-analysis [16], [17]. If evidence of an interaction or difference in the size of effect was found in a particular subgroup, we assessed whether a similar meta-analysis on PFS, a potentially more sensitive outcome, would support or refute the findings.

All p-values are two-sided. All analyses were carried out using Stata, version 14.2.

## 3 Results

**Fig. 1:**
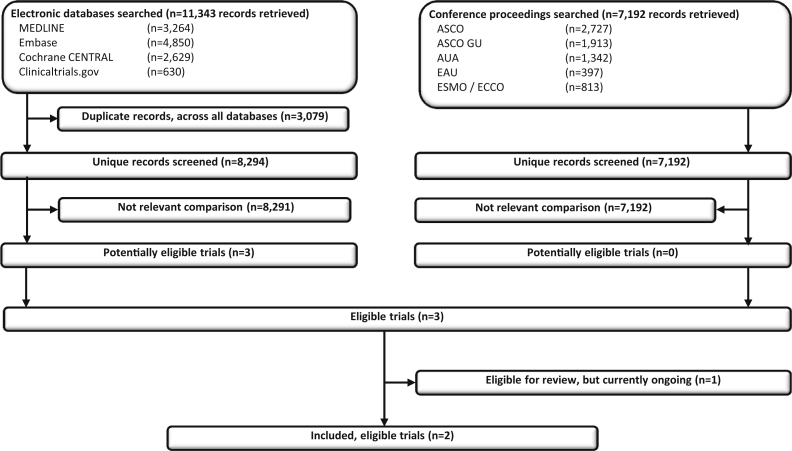
**PRISMA flow diagram of trial identification, screening, eligibility and inclusion**. ASCO, American Society of Clinical Oncology; AUA, American Urological Association; EAU, European Association of Urology; ECCO, European Cancer Organisation; ESMO, European Society for Medical Oncology; PRISMA, Preferred Reporting Items for Systematic Reviews and Meta-analyses.

Our broad searches for all trials in mHSPC retrieved 15,486 unique records, and we identified three trials eligible for this particular review (Fig. 1). Two trials (LATITUDE and STAMPEDE) compared AAP plus ADT with ADT [11], [12]; one of these (STAMPEDE) as part of a multi-arm, multi-stage design [18]. Both have recently published results (Table 1) [11], [12]. Although STAMPEDE includes men with both metastatic and non-metastatic disease [12], we obtained information and results for the patients with metastatic disease. The third (PEACE-1) is a factorial trial investigating the addition of AAP and/or RT to ADT and is still accruing patients (Table 1). Moreover, the PEACE-1 protocol was amended in 2015 to allow docetaxel in all arms, and since then, approximately two-thirds of randomised men have received docetaxel in addition to ADT, with one-third receiving ADT without docetaxel. Thus, we have been able to include results for two trials [11], [12], which, accounting for the amended PEACE-1 protocol, represents 82% (2201/2677) of all men randomised to AAP plus ADT versus ADT (without docetaxel in either arm), a higher percentage than originally anticipated.

LATITUDE and STAMPEDE randomised men with mHSPC between 2011 and 2014. In both trials, abiraterone acetate was administered as a single dose of 1000 mg per day together with prednisolone or prednisone (5 mg daily) to prevent secondary mineralocorticoid excess, until disease progression, withdrawal of consent or unacceptable toxicity. Median follow-up was 30 months in LATITUDE and 41 months for men with metastatic disease in STAMPEDE [11], [12]. Based on randomisation sequence generation, allocation concealment, blinding, completeness of outcome data and selective outcome reporting, both trials were judged to have a low risk of bias (Table 2).

**Table 2:** Assessment of risk of bias (based on overall survival).

Trial ID	Adequate sequence generation	Allocation concealment	Masking	Incomplete outcome data addressed	Free of selective reporting
STAMPEDE [12]	Central randomisation using a computerised algorithm. A minimisation method with a random element of 80% was used to stratify for a number of clinically important factors	Central telephone randomisation	Open label; blinding to treatment allocation considered impractical and of limited value, given the primary outcome of death from any cause	All randomised patients included in analyses	All outcomes of interest reported
LATITUDE [11]	A computer-generated randomisation schedule was used. Country by country randomisation was performed using permuted block randomisation.	Centralised interactive Web response system (IWRS)	Double blind, placebo controlled. Participants, care-givers and investigators unaware of treatment allocation	All randomised patients included in analyses	All outcomes of interest reported

All men in LATITUDE and 94% of men in STAMPEDE were classed as newly diagnosed with mHSPC [11], [12] and were receiving long-term ADT for the first time, the remainder having relapsed after prior treatment for localised disease. Most received LHRH-based therapy (≈86%) rather than orchiectomy. Across the two trials, men were aged between 33 and 92 years (LATITUDE median 67 years [interquartile range (IQR) 61–73 years]; STAMPEDE median 67 years [IQR 62–71 years]) mostly with a Gleason sum score of ≥8 (87%), good performance status (97% Eastern Cooperative Oncology Group/World Health Organisation 0–1) and positive pelvic nodes (52%) [11], [12]. The notable differences between the trials were that LATITUDE included more men with a performance status of 1 (42% versus 24% in STAMPEDE), and with visceral, soft tissue and nodal metastases (65% versus 35% in STAMPEDE) with or without bone metastases [11], [12]. Furthermore, as a Gleason sum score of ≥8 was one of the eligibility criteria for the LATITUDE trial [11], relatively few men with a Gleason score of <8 were included (2% versus 23% in STAMPEDE) (Table 3). Therefore, men in the LATITUDE trial were generally higher risk patients with a greater burden of disease. The LATITUDE trial results are from an interim analysis conducted when approximately 50% of expected death events had occurred, but after unanimous approval by the Independent Data Monitoring Committee, the trial was unblinded and the analysis is considered final [11].

**Table 3:** Characteristics of included patients.

	STAMPEDE		LATITUDE	
	ADT	ADT + AAP	ADT	ADT + AAP
Number of patients	502	500	602	597
**Age**				
Median (IQR)	67 (62–72)	67 (62–71)	67 (61–73)	68 (61–73)
Range	39–84	42–85	33–92	38–89
**PSA [ng/ml]**				
Median (IQR)	97 (26–358)	96 (29–371)	23.05 (4.96–112.66)	25.43 (4.62, 117.58)
Range	0–10530	0–21460	(0.1–8889.6)	(0–87775.9)
**Time from initial diagnosis**^a^				
Median	2.3	2.5	2.0	1.8
Range	0–160	0–177	(0–4)	(0–3)
Missing	1	3	0	0
**WHO PS (ECOG PS)**				
0	370 (73.7%)	374 (74.8%)	331 (55.0%)	326 (54.6%)
1	125 (24.9%)	119 (23.8%)	255 (42.4%)	245 (41.0%)
2	7 (1.4%)	7 (1.4%)	16 (2.7%)	26 (4.4%)
**T category**^b^				
T0	1 (0.2%)	2 (0.4%)	1 (0.2%)	0
T1	10 (2.0%)	5 (1%)	25 (4.2%)	29 (4.9%)
T2	45 (9.0%)	44 (8.8%)	113 (18.8%)	94 (15.8%)
T3	270 (53.8%)	288 (57.6%)	254 (42.3%)	246 (41.3%)
T4	137 (27.3%)	118 (23.6%)	128 (21.3%)	159 (26.7%)
Tx	39 (7.8%)	43 (9.2%)	80 (13.3%)	68 (11.4%)
**N category**^c^				
N0	175 (34.9%)	167 (33.4%)	151 (25.2%)	152 (25.5%)
N+	291 (58.0%)	292 (58.4%)	280 (46.7%)	280 (47.0%)
Nx	36 (7.2%)	41 (8.2%)	169 (28.2%)	164 (27.5%)
**Location of metastases**				
Bone	448 (89.2%)	434 (86.8%)	585 (97.5%)	580 (97.3%)
Liver	8 (1.6%)	7 (1.4%)	30 (5.0%)	32 (5.4%)
Lung	21 (4.2%)	21 (4.2%)	72 (12.0%)	73 (12.2%)
Nodal	150 (29.9%)	142 (28.4%)	287 (47.8%)	283 (47.5%)
Other	26 (5.2%)	23 (4.6%)	182 (30.4%)	180 (30.1%)
**Disease history (newly diagnosed/relapsed)**				
Newly diagnosed M1	476 (94.8%)	465 (93%)	602 (100%)	597 (100%)
Previously treated M1	26 (5.2%)	35 (7.0%)	0	0
**Gleason sum**				
≤7	119 (23.7%)	115 (23%)	16 (2.7%)	13 (2.2%)
8–10	373 (74.3%)	364 (72.8%)	586 (97.3%)	584 (97.8%)
Unknown	10 (2.0%)	21 (4.2%)	0	0
**Type of ADT**^d^				
Orchiectomy	3 (0.6%)	3 (0.6%)	71 (11.8%)	73 (12.2%)
Bicalutamide/anti-androgen alone	1 (0.2%)	0	84 (14.0%)	46 (7.7%)
Dual androgen blockade	3 (0.6%)	1 (0.2%)	NA	NA
LHRH based	495 (98.6%)	496 (99.2%)	450 (74.8%)	449 (75.2%)

AAP, abiraterone acetate plus prednisone/prednisolone; ADT, androgen deprivation therapy; ECOG, Eastern Cooperative Oncology Group; LHRH, luteinising hormone–releasing hormone; PS, performance score; WHO, World Health Organisation.

a For STAMPEDE, this also includes men who have relapsed after previous radical treatment.

b In LATITUDE, T category unaccounted for in one patient from each arm.

c In LATITUDE, N category unaccounted for in two patients in ADT arm and one patient in ADT + AAP.

d In LATITUDE, in ADT arm, some patients may have received anti-androgen in addition to LHRHa-based treatment; the patients unaccounted for in ADT + AAP may not yet have been started on ADT as diagnosed only very recently.

**Fig. 2:**
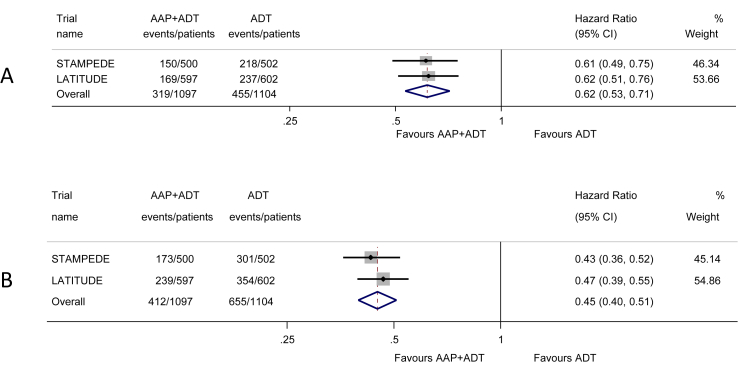
**Effect of adding AAP to ADT on (A) overall survival and (clinical/radiological) progression-free survival (B) in men with mHSPC**. Each filled square denotes the HR for that trial comparison, with the horizontal lines showing the 95% CI. The size of the square is directly proportional to the amount of information contributed by a trial. The diamond represents a (fixed-effect) meta-analysis of the trial HRs, with the centre of this diamond indicating the HR and the extremities the 95% CI. AAP, abiraterone acetate plus prednisone/prednisolone; ADT, androgen deprivation therapy; CI, confidence interval; HR, hazard ratio; mHSPC, metastatic hormone-sensitive prostate cancer.

For the primary outcome of OS, results were based on all 2201 men with metastatic disease from the two trials and included 774 deaths. Median OS in LATITUDE is 34.7 months in the ADT arm, but it has not yet been reached in the AAP arm. In STAMPEDE, median OS is 48 months in the ADT arm and again has not been reached in the AAP arm. Based on these data, we found a highly significant 38% reduction in the risk of death (HR = 0.62, 95% CI = 0.53–0.71, p = 0.55 × 10^−10^; Fig. 2). Applying the HR to the average control-group survival from LATITUDE and STAMPEDE [11], [12], translates to a 14% absolute improvement in OS at 3 years with AAP, from 55% to 69%. The results across trials were remarkably consistent, and there was no evidence of statistical heterogeneity (Heterogeneity chi^2^ = 0.01, df = 1, p = 0.90, I^2^ = 0%).

The secondary outcome of PFS was defined differently in each of the two trials. In LATITUDE [11], this was defined as the time to radiologically confirmed progression or death by any cause, whereas in STAMPEDE, it was defined as the time to first symptomatic clinical (defined as new cancer-related symptoms) or radiological progression or death from prostate cancer [12]. Despite these differences, we felt that the outcomes were sufficiently compatible to combine. Results were again available for all 2201 patients and included 1067 events. Median PFS in LATITUDE is 14.8 months in the ADT arm and 33 months in the AAP arm. In STAMPEDE, median PFS is 24 months in the ADT arm and has not been reached in the AAP arm. Based on these data, we observed a highly significant 55% reduction in the risk of clinical/radiological PFS (HR = 0.45, 95% CI = 0.40–0.51, p = 0.66 × 10^−36^; Fig. 2). Applying the HR to the average control-group PFS from LATITUDE and STAMPEDE translates to a 28% absolute improvement in PFS at 3 years with AAP, from 30% to 58%. Although PFS was differently defined across the two trials, individual trial results were very consistent, with no evidence of statistical heterogeneity (Heterogeneity chi^2^ = 0.40, df = 1, p = 0.53, I^2^ = 0%). We were unable to assess FFS, as only one of the trials (STAMPEDE) analysed this outcome [12].

**Fig. 3:**
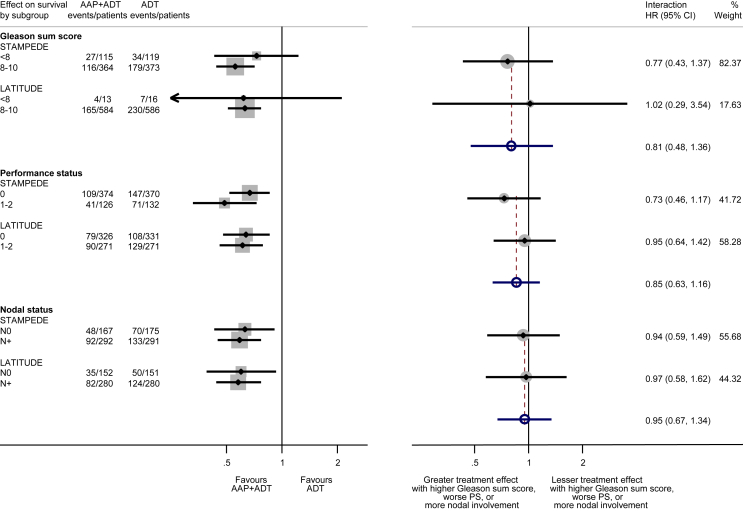
**Effect of adding AAP to ADT on overall survival by nodal status, Gleason sum score and performance status**. Each filled square denotes the HR for each subgroup of men defined by, Gleason sum score, nodal status and PS within each trial, with the horizontal lines showing the 95% CI. The size of the square is directly proportional to the amount of information contributed by a subgroup. Each filled circle denotes the HR for the interaction between the effect of chemotherapy and these subgroups for each trial, with the horizontal lines showing the 95% CI. The size of each circle is directly proportional to the amount of information contributed by a trial. The open circle represents a (fixed-effect) meta-analysis of the interaction HRs, with the horizontal line showing the 95% CI. AAP, abiraterone acetate plus prednisone/prednisolone; ADT, androgen deprivation therapy; CI, confidence interval; HR, hazard ratio; PS, performance status.

**Fig. 4:**
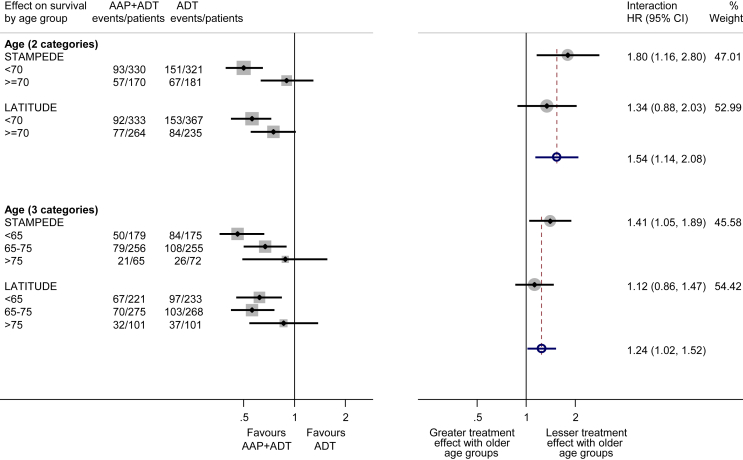
**Effect of adding AAP to ADT on overall survival by age group**. Labelling and conventions as in Fig. 3. AAP, abiraterone acetate plus prednisone/prednisolone; ADT, androgen deprivation therapy; CI, confidence interval; HR, hazard ratio.

As most men were newly diagnosed and received LHRH-based ADT, there was no value in conducting subgroup analyses by disease history and type of ADT. Likewise, for results based on location of metastases, clear overlap between sites of metastases meant that these could not be meaningfully combined in a meta-analysis. We did not observe any variation in the effect of treatment on OS by Gleason sum score (interaction HR = 0.81, 95% CI = 0.48–1.36, p = 0.42), performance status (interaction HR = 0.85, 95% CI = 0.63–1.16, p = 0.31) or nodal status (interaction HR = 0.95, 95% CI = 0.67–1.34, p = 0.77; Fig. 3). For the outcome of OS, there was evidence that the size of benefit was greater in younger men and less pronounced in older men, both when age groups were defined as in the STAMPEDE trial (<70, ≥70: interaction HR = 1.54, 95% CI = 1.14–2.08, p = 0.005) [12] and when the categories were amended to achieve a broader distribution of men across age groups (<65, 65–75, >75: interaction HR = 1.24, 95% CI = 1.02–1.52, p = 0.033; Fig. 4). However, this pattern was less clear when based on PFS (<70, ≥70: interaction HR = 1.30, 95% CI = 1.00–1.69, p = 0.05; <65, 65–75, >75: interaction HR = 1.15, 95% CI = 0.95–1.38, p = 0.14; Web Fig. 1).

**Fig. 5:**
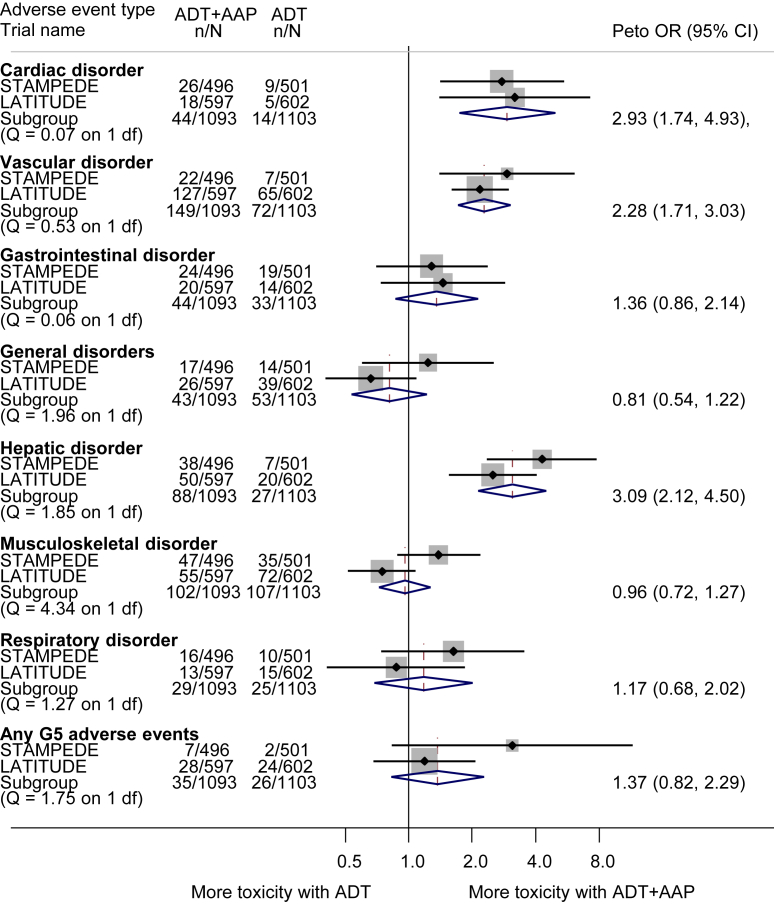
**Effect of adding AAP to ADT on grade III–IV and grade V adverse events**. Apart from a Peto OR (rather than hazard ratio) measure of effect, labelling and conventions are as in Fig. 2. AAP, abiraterone acetate plus prednisone/prednisolone; ADT, androgen deprivation therapy; CI, confidence interval; OR, odds ratio.

We obtained all available grade III–IV toxicity data, from both trials, and across all categories, but it should be noted that the majority of these were grade III toxicities. Although both trials used the Common Terminology Criteria for Adverse Events (CTCAE, version 4.0), there was some variation in the types of events underpinning the main toxicity categories. Where these were deemed sufficiently similar across trials (musculoskeletal, gastrointestinal, respiratory and general disorders), results were combined in meta-analysis. However, as LATITUDE analysed cardiac and vascular toxicities separately, whereas these were combined in STAMPEDE, the STAMPEDE team provided additional results for cardiac and vascular toxicities separately [11], [12]. Similarly, the LATITUDE team provided further results to facilitate pooling of hepatic toxicity as defined in the STAMPEDE trial [11], [12]. We were unable to combine other grade III–IV toxicities that had been observed in considerable numbers in the individual trials (e.g. endocrine, metabolic disorders and nervous system disorders) in meta-analysis [11], [12]. Overall, we found no increase in grade III–IV musculoskeletal, gastrointestinal, respiratory or general disorders with the addition of AAP (Fig. 5). However, there was an approximate three-fold increase in grade III–IV acute cardiac (Peto OR = 2.93, 95% CI 1.74–4.93, p < 0.001) and hepatic toxicity (Peto OR = 3.09, 95% CI 2.12–4.50, p < 0.001) and an approximate two-fold increase in grade III–IV vascular events (OR = 2.28, 95% CI 1.71–3.03, p < 0.001), the majority of which (≥90%) were related to hypertension.

Across the two trials, there were 61 deaths associated with grade V adverse events but no clear evidence that these were increased with the addition of AAP (Peto OR = 1.37 95% CI 0.82–2.29, p = 0.23; Fig. 5).

## 4 Discussion

### 4.1 Summary of results

We have shown that adding AAP to ADT provides highly significant and substantial reductions in the risk of both death (38%) and clinical/radiological PFS (55%) for men with mHSPC. These translate into 14% and 28% absolute improvements in OS and PFS, respectively, at 3 years after randomisation. The OS benefit did not vary by Gleason sum score, performance status or nodal status. Although our results suggest that the observed survival benefit may be greater in younger men and lesser in older men, the sample size in this latter group is small. In addition, for PFS there was less evidence that the treatment effect varied with age. Based on the data available, acute grade III–IV cardiac, hepatic and to a lesser degree vascular toxicities, were increased with the use of AAP. There was no statistically significant excess of deaths associated with use of AAP.

### 4.2 Strengths

This is the first systematic review of adding AAP to ADT in the mHSPC setting and includes data on 2201 men from two large trials [11], [12], representing 82% of all men randomised to ADT plus AAP versus ADT (without docetaxel in either arm). In addition, results across trials are very clear, highly consistent and allow us to provide very precise estimates of the direction and size of effects. Therefore, these meta-analysis results provide reliable and robust evidence to guide practice and future research. Using our collaborative FAME approach, we have been able to synthesise the effects of adding AAP to ADT in a more timely, reliable, and meaningful manner than is usually possible with aggregate data [7]. We were able to identify all eligible trials, in advance of results being available, anticipate when results of STAMPEDE and LATITUDE were due to emerge and obtain up-to-date accrual information for the ongoing PEACE-1 trial [11], [12]. The STOPCaP Project Management Group also gained access to pre-publication results and additional unreported analyses. We obtained additional PFS results for men with metastatic disease from STAMPEDE, allowing us to examine the consistency of effect across the two trials and provide the best estimate of the sizes of the effect. By obtaining subgroup analyses based on STAMPEDE M1 patients and additional analyses of LATITUDE, we were also able to investigate whether the effects of AAP on OS and PFS are consistent across different types of men, with far greater power than either individual trial. By collecting acute toxicity and, where possible, harmonising categories, we have been able to provide the first formal analysis of grade III, IV and grade V adverse events within and across trials. This has confirmed that there is no increase in deaths, but shown serious cardiac, vascular and hepatic adverse events are exacerbated with AAP. In addition, the FAME approach has also allowed us to publish the review results in a similar time frame to the individual trial results [11], [12].

### 4.3 Limitations

As stated earlier, the LATITUDE trial results are from a planned interim analysis, but after approval by the Independent Data Monitoring Committee, are now considered final [11]. The effect sizes observed in trials that report early can lessen with longer follow-up durations; however, the effect seen in LATITUDE showed internal consistency across planned subgroup analyses and is substantial and highly significant in its own right [11]. Moreover, the results are consistent with the final results of STAMPEDE [12], which were reported at the pre-planned time. Although in LATITUDE, PFS was defined as the time to radiologically confirmed progression or death by any cause [11] and in STAMPEDE as the time to first symptomatic clinical or radiological progression or death from prostate cancer [12], again, results were very consistent. The apparent difference in the size of the OS benefit by age may reflect that older men are at higher risk of dying from other co-existing conditions or are less able to tolerate treatments. However, to establish definitively whether the effect of AAP varies with age will require an analysis of age as a continuous variable, which affords greater power but necessitates the collection of IPD.

Notably, all men recruited to LATITUDE and the majority of men with metastatic disease recruited to STAMPEDE had newly diagnosed disease [11], [12]. Therefore, while we can be certain of the treatment benefit in these men, there remains some uncertainty about whether the benefits of AAP can be extrapolated to men who have relapsed after prior local treatment for localised disease. Furthermore, in LATITUDE [11], all patients had high-risk metastatic disease, and most also had a high burden (or volume) or disease while characterisation of risk or burden of disease in STAMPEDE is unknown. The retrospective assessment of disease volume in STAMPEDE, combined with the planned collection of IPD from the AAP and docetaxel trials in mHSPC, may therefore help to determine whether effects vary by disease volume [12]. Results of the PEACE-1 trial (Table 1) will later add to the weight of evidence about the effects of AAP, but only results for men who did not receive docetaxel will be eligible for inclusion in this current comparison (expected to be ≈476/2677; 18%), so would be unlikely to materially affect our findings. In addition, these results are unlikely to be available before 2020.

### 4.4 Context

Based on the current and a prior review [10], and assuming control-group survival of 55%, AAP provides a 14% absolute improvement in 3-year OS compared to an 8% absolute improvement with docetaxel. This crude comparison does not take into account the different time frames and patient populations across these trials. An increase in grade III–IV adverse events was observed in both the abiraterone and docetaxel trials, most commonly neutropenia with docetaxel and cardiovascular or hepatic toxicity with abiraterone. However, in both sets of trials, the incidence of treatment-related deaths was relatively low. While not a formally powered comparison, only the multi-arm STAMPEDE trial can directly compare the effects of AAP plus ADT with the effects of docetaxel plus ADT, although this data is not yet available. A network meta-analysis (NMA) that makes use of this and all other available direct and indirect comparisons of current therapies for mHSPC may make it possible to rank all the relative effects, with the relative benefits of AAP and docetaxel being of particular interest. This NMA is being developed collaboratively as part of the wider STOPCaP project.

Given the improved OS seen with both AAP and docetaxel, together with their differing mechanisms of action, a major question is also whether the effects of these two agents are additive. This will take some years to assess, as only the second phase of the PEACE-1 trial will provide data on ADT plus docetaxel plus AAP. Therefore, we would actively encourage participation to this trial. Furthermore, as enzalutamide has been shown to have a similar clinical effect on androgen signalling in castration-resistant prostate cancer as AAP, the ENZAMET trial (NCT02446405) of enzalutamide plus ADT [19], which has recently completed accrual, and the ARCHES trial (NCT02677896) which is still recruiting [20], both of which are stratified by docetaxel use, will further augment our knowledge of such ‘triplet therapy’. Also, the ARASENS trial (NCT02799602) will provide evidence about the effects of darolutamide in men receiving ADT plus docetaxel as their standard of care [21]. However, results of ARCHES and ARASENS are unlikely to be available in the near future.

### 4.5 Implication(s) of findings

Until evidence about the relative effects of adding AAP or docetaxel to ADT, or the combination, becomes available, physicians are likely to have to choose between AAP and docetaxel. They will need to take account of efficacy, toxicity and tolerability, and ease of administration of AAP compared with docetaxel, as well as cost and access to these agents. A similar choice will likely need to be considered as part of the ongoing STAMPEDE comparisons. The collection of IPD from all relevant trials, as part of the STOPCaP collaboration, will be required to determine definitively if particular men (for example older or younger) may benefit more or less from these and other emerging treatments for mHSPC, to either target effective treatments appropriately or make them more widely available. IPD will also be valuable for tackling other important clinical and scientific questions arising, including the identification of surrogate outcomes by building on the Intermediate Clinical Endpoints in Cancer of the Prostate (ICECaP) initiative in non-metastatic prostate cancer [24]. Therefore, we are developing the STOPCaP/ICECaP M1 repository of contemporary trials in mHSPC. This work will be supported by the MRC (MC_UU_12023/25) and a Prostate Cancer UK Research Innovation Award (RIA16-ST2-020).

### 4.6 Conclusion

Adding AAP to ADT is a highly effective treatment option for men with mHSPC and offers an alternative to docetaxel, for men who are starting treatment for the first time. Future research will need to address which of these two agents or whether their combination is most effective, and for whom.

## Contributors

LHMR, JFT, SB and CLV comprised the Project Management Group and were responsible for the day-to-day running of the project. They conceived the project and developed the protocol, which guided both data collection and analysis, with help and advice from MDM, MKBP and CJS (the International Advisory Group) in advance of any results being known. LHMR and SB extracted the data. LHMR, SB and CLV performed all statistical analyses. The article was drafted by LHMR, JFT, SB and CLV and then circulated to all authors for comments.

KF, TK, BM and NT represented the LATITUDE trial and supplied pre-publication information and results. NDJ, MSp, MSy and NWC represented the STAMPEDE trial and supplied pre-publication information and results. All authors contributed to the interpretation of the results and critically revised each draft.

## Role of the funding source

The funder of the study (UK Medical Research Council) had no role in study design, data collection, data analysis, data interpretation or writing of the report. The corresponding author had full access to all the data in the study and had final responsibility for the decision to submit for publication.

## Conflict of interest statement

LHMR, JFT, SB and CLV declare no competing interests. MKBP, MSp and MSy report grants from Astellas, Janssen, Novartis, Pfizer, Sanofi and Clovis Oncology. NWC reports participation on advisory boards for Astellas, Janssen, Ferring, Bayer, AstraZeneca and corporate-sponsored research for AstraZeneca. KF reports participation on advisory boards/honorarium for Amgen, Astrazeneca, Astellas, Bayer, Clovis, Janssen, MSD, Orion, Curevac, Sanofi and Genentech. BM, NT and TK are all employees of Janssen. MDM reports delivering a paid lecture for Janssen and advisory board membership for Sanofi and Bayer. NDJ reports grants, personal fees and non-financial support from Janssen, Astellas, Sanofi, Novartis grants and non-financial support from Clovis Oncology and Pfizer. CJS reports stock ownership in BIND; advisory board participation for Astellas/Medivation, Astra Zeneca, Sanofi, Janssen, BIND and Bayer; grants from Janssen, Astellas/Medivation, Sanofi, Janssen, Soti and Exelixis and consultancy for Astellas/Medivation, Pfizer, Sanofi, Janssen, BIND, Bayer and Genentech.

## Appendix A Supplementary data

Web Figure 1 Effect of adding AAP to ADT on progression-free survival by age group. Each filled square denotes the hazard ratio (HR) for each subgroup of men defined by, Gleason sum score, nodal status and performance status within each trial, with the horizontal lines showing the 95% confidence interval (CI). The size of the square is directly proportional to the amount of information contributed by a subgroup. Each filled circle denotes the HR for the interaction between the effect of chemotherapy and these subgroups for each trial, with the horizontal lines showing the 95% CI. The size of each circle is directly proportional to the amount of information contributed by a trial. The open circle represents a (fixed-effect) meta-analysis of the interaction HRs, with the horizontal line showing the 95% CI. Web Figure 1
