# Supplementary material for: Adding abiraterone to androgen deprivation therapy in men with metastatic hormone-sensitive prostate cancer: A systematic review and meta-analysis
Source: Eur J Cancer. 2017 Oct;84:88–101. doi: 10.1016/j.ejca.2017.07.003 (PMC5630199; doi:10.1016/j.ejca.2017.07.003)
Supplement: Web Figure 1 — Effect of adding AAP to ADT on progression-free survival by age group. Each filled square denotes the hazard ratio (HR) for each subgroup of men defined by, Gleason sum score, nodal status and performance status within each trial, with the horizontal lines showing the 95% confidence interval (CI). The size of the square is directly proportional to the amount of information contributed by a subgroup. Each filled circle denotes the HR for the interaction between the effect of chemotherapy and these subgroups for each trial, with the horizontal lines showing the 95% CI. The size of each circle is directly proportional to the amount of information contributed by a trial. The open circle represents a (fixed-effect) meta-analysis of the interaction HRs, with the horizontal line showing the 95% CI. [file mmc2.pptx]

## Slide 1
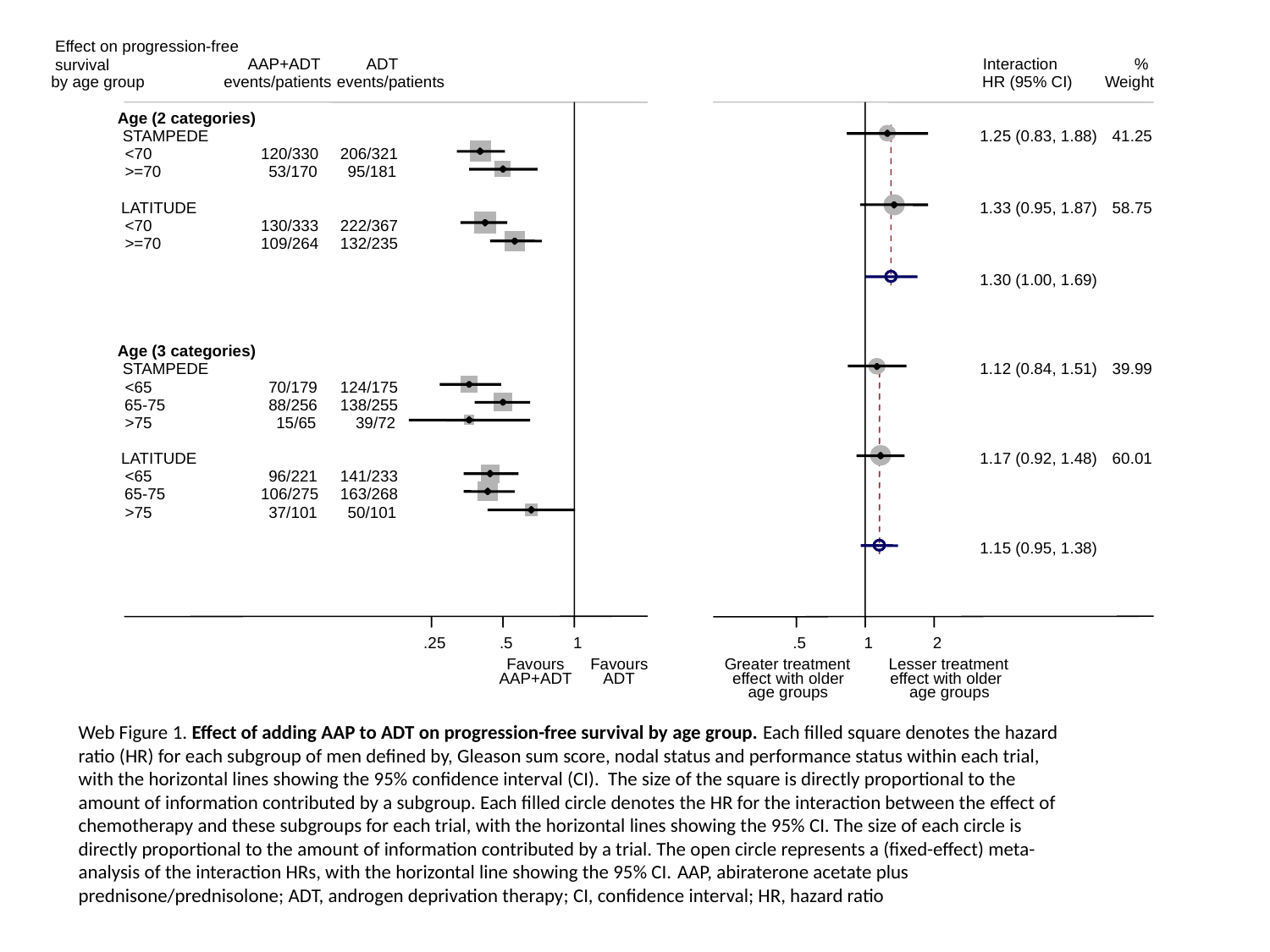

Effect on progression-free
survival
AAP+ADT
ADT
by age group
events/patients
events/patients
Age (2 categories)
STAMPEDE
<70
120/330
206/321
>=70
53/170
95/181
LATITUDE
<70
130/333
222/367
>=70
109/264
132/235
Age (3 categories)
STAMPEDE
<65
70/179
124/175
65-75
88/256
138/255
>75
15/65
39/72
LATITUDE
<65
96/221
141/233
65-75
106/275
163/268
>75
37/101
50/101
.25
.5
1
Favours
Favours
AAP+ADT
ADT
Interaction
%
HR (95% CI)
Weight
1.25 (0.83, 1.88)
41.25
1.33 (0.95, 1.87)
58.75
1.30 (1.00, 1.69)
1.12 (0.84, 1.51)
39.99
1.17 (0.92, 1.48)
60.01
1.15 (0.95, 1.38)
.5
1
2
Greater treatment
Lesser treatment
effect with older
effect with older
age groups
age groups
Web Figure 1. Effect of adding AAP to ADT on progression-free survival by age group. Each filled square denotes the hazard ratio (HR) for each subgroup of men defined by, Gleason sum score, nodal status and performance status within each trial, with the horizontal lines showing the 95% confidence interval (CI). The size of the square is directly proportional to the amount of information contributed by a subgroup. Each filled circle denotes the HR for the interaction between the effect of chemotherapy and these subgroups for each trial, with the horizontal lines showing the 95% CI. The size of each circle is directly proportional to the amount of information contributed by a trial. The open circle represents a (fixed-effect) meta-analysis of the interaction HRs, with the horizontal line showing the 95% CI. AAP, abiraterone acetate plus prednisone/prednisolone; ADT, androgen deprivation therapy; CI, confidence interval; HR, hazard ratio
